# Secular trends in age at menarche among Chinese girls from 24 ethnic minorities, 1985 to 2010

**DOI:** 10.3402/gha.v8.26929

**Published:** 2015-07-27

**Authors:** Yi Song, Jun Ma, Anette Agardh, Patrick W.C. Lau, Peijin Hu, Bing Zhang

**Affiliations:** 1 Institute of Child and Adolescent Health, School of Public Health, Peking University, Beijing, China; 2 Social Medicine and Global Health, Department of Clinical Sciences, Lund University, Malmö, Sweden; 3 Department of Physical Education, Hong Kong Baptist University, Kowloon Tong, Hong kong, People's Republic of China

**Keywords:** menarche, puberty development, China, girls, ethnic minorities, trend analysis

## Abstract

**Background:**

Declining age at menarche has been observed in many countries. In China, a decrease of 4.5 months per decade in the average age at menarche among the majority Han girls has recently been reported. However, the trends in age at menarche among ethnic minority girls over the past 25 years remain unknown.

**Objectives:**

To compare the differences in median age at menarche among girls aged 9–18 years across 24 ethnic minorities in 2010 and to estimate the trends in age at menarche in different ethnic minorities from 1985 to 2010.

**Design:**

We used data from six cross-sectional Chinese National Surveys on Students’ Constitution and Health (1985, 1991, 1995, 2000, 2005, and 2010). The median age at menarche was estimated by using probit analysis.

**Results:**

In 2010, the ethnic minorities with the earliest age at menarche were the Koreans (11.79 years), Mongolians (12.44 years), and Zhuang (12.52 years). The three ethnic minorities with the latest age at menarche were the Sala (14.32 years), Yi (13.74 years), and Uighurs (13.67 years). From 1985 to 2010, the age at menarche declined in all 24 minority groups. The Lisu, Kazakh, and Korean minorities showed the largest reductions in age at menarche by 1.79 (*p<*0.05), 1.69 (*p<*0.05), and 1.57 (*p<*0.05) years, respectively, from 1985 to 2010. The Yi, Sala, and Li minorities showed the smallest reductions, with age at menarche declining by only 0.06 (*p>*0.05), 0.15 (*p>*0.05), and 0.15 (*p>*0.05) years, respectively, in the same period.

**Conclusion:**

A large variation in age at menarche was observed among different ethnic minorities, with the earliest age at menarche found among Korean girls. A reduction in the average age at menarche appeared among most of the ethnic minorities over time, and the largest decrease was observed in Lisu, Kazakh, and Korean girls. Thus, health education should focus on targeting the specific needs of each ethnic minority group.

The onset of menarche is an important milestone in female sexual maturation. Many international studies have shown that puberty is occurring earlier among girls now than in previous decades (1–4). In Europe, the median age at menarche decreased by 2 to 3 months per decade from 16.5 years in 1840 to about 13.0 years in the 1960s, with a variation of 0.5 years between countries (5–7). In the United States, the average age at menarche decreased from 12.75 years between 1963 and 1970 to 12.54 years between 1988 and 1994, and subsequently to 12.34 years between 1999 and 2002 (8, 9). In China, the average age at menarche declined from 13.41 to 12.47 years between 1985 and 2010 (10). The age at menarche of a population may reflect the timing of sexual maturation, population nutritional status, and socio-economic conditions (11, 12). If the age at menarche is earlier than the norm, it can pose a health risk. Age at menarche may affect the risk of various diseases in adulthood; for example, early menarche may be associated with obesity and cardiovascular disease, as well as certain cancers (13–15). Reports of age at menarche among Chinese girls have been sparse, and many are outdated or focus only on girls of Han ethnicity. National data on the timing of menarche among ethnic minority girls is not available, and the trends in age at menarche among ethnic minority girls over the past 25 years are unknown.

The majority of the population in China belongs to the Han ethnic group, and the other 55 ethnic groups are referred to as *ethnic minorities*. Although they are in the minority, the absolute number of an ethnic minority in China may be larger than in any other country or region. According to the sixth Chinese national census, conducted in 2010, 113,792,211 (8.49%) of the country's total population belonged to ethnic minorities (16). For example, there are nearly 9 million Mongolians in the world, with approximately 5.8 million living in China, 2.4 million in the People's Republic of Mongolia, 0.5 million in Russia, and the remainder in other countries, such as Afghanistan, Pakistan, and Iran (17). As a multi-ethnic country, China provides a good opportunity to describe the diversity in age at menarche among different ethnic minorities during the same period. It is currently unknown whether age at menarche has decreased among all ethnic minority girls and whether age at menarche among girls from the same ethnic minority living in different regions such as urban or rural areas shows similar secular trends. Likewise, it is unknown whether age at menarche among ethnic minorities who also live in other countries or regions, such as Mongolians, Uighurs, Koreans, Tibetans, Kazakhs, Miao, and Yao, shows similar secular trends.

Data concerning menarche among ethnic minority girls are available from the Chinese National Surveys on Students’ Constitution and Health (CNSSCH) (18–23), which have been conducted every 5 years since 1985 under the combined auspices of the Ministry of Education, the Ministry of Health, the Ministry of Science and Technology, the State of National Affairs, and the State Sports General Administration of the People's Republic of China. As a national sample of school-age children in China, it includes both Han and ethnic minority students and thus provides an opportunity to study the trends regarding age at menarche among ethnic minorities. The present analysis sought to 1) compare the differences of average age at menarche among 24 ethnic minorities in 2010 and 2) estimate the trends of age at menarche among different ethnic minorities from 1985 to 2010.

## Subjects and methods

### Subjects

**Fig. 1:**
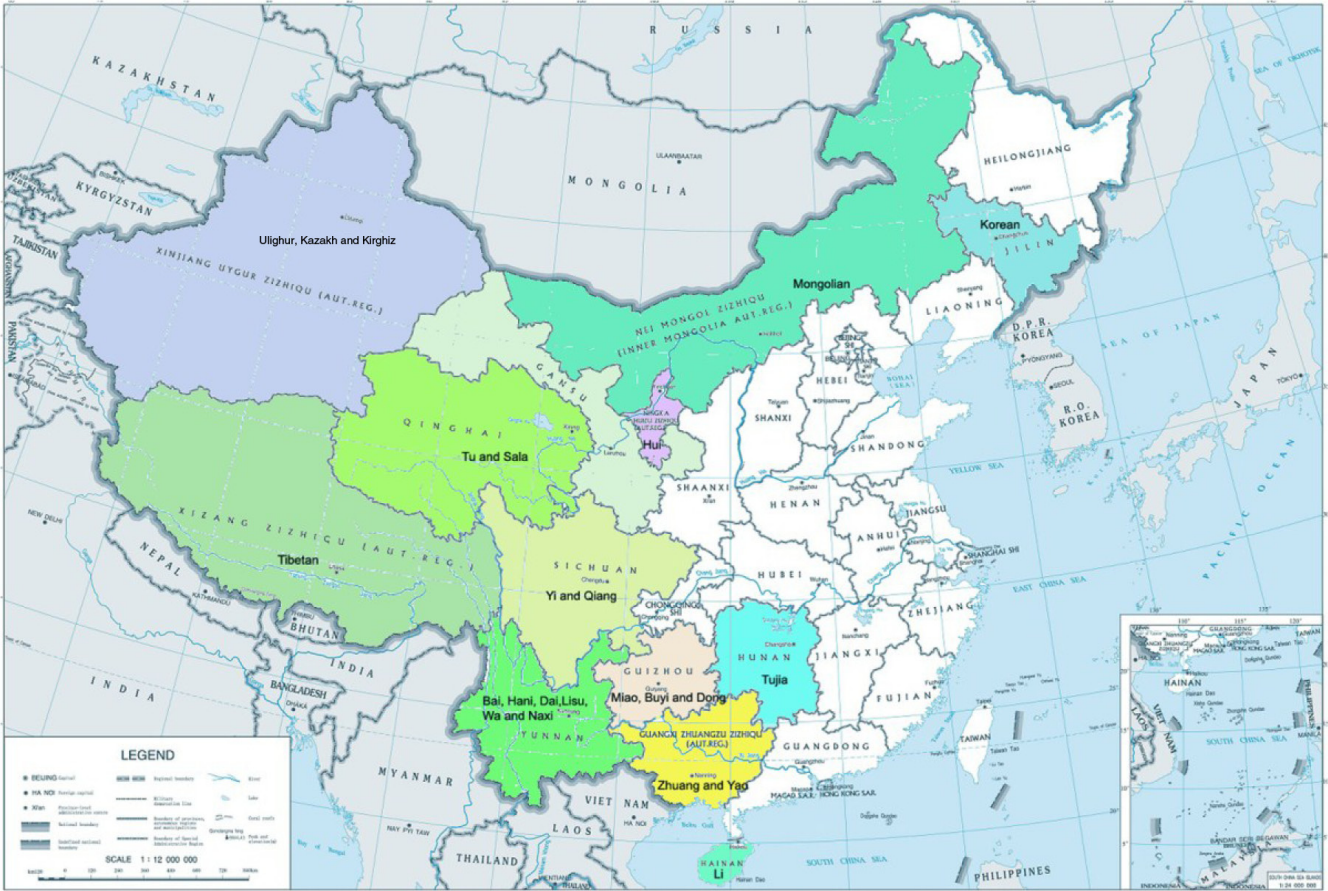
The locations of 24 ethnic minorities sampled in China.

Data were obtained from the 1985, 1991, 1995, 2000, 2005, and 2010 cycles of the CNSSCH (18–23). The CNSSCH is the largest nationally representative sample of more than 200,000 school-aged (6–22 years) children in China in each survey point, among whom more than 90% of the subjects are of Han nationality. The present study included only girls aged 9–18 years from the following 24 ethnic minorities, who were mainly sampled in the ethnic minority autonomous region or prefecture: Mongolian, Hui, Uighur, Zhuang, Korean, Tibetan, Miao, Yi, Buyi, Dong, Yao, Bai, Tujia, Hani, Kazakh, Dai, Li, Lisu, Wa, Naxi, Kirghiz, Tu, Qiang, and Sala (Fig. 1). Only the Mongolian, Hui, Uighur, Zhuang, and Korean minorities were sampled in urban and rural areas. The data from the six cycles of the CNSSCH were checked for comparability and reliability of these three indicators: 1) all the subjects and their parents were of the same ethnic origin and had lived in the local areas for at least 1 year; 2) all subjects were uniformly measured in the same year using the same methods and in the same way; 3) all subjects had a thorough medical examination before measurement and were generally healthy and free from overt disease or physical/mental deformities. The sample sizes of the various ethnic minorities at different survey points ranged from 224 to 2,400 (Table 1); the sample size of each survey ranged from 21,382 to 31,711, and the total sample size (all surveys) was 149,482. The project was approved by the Medical Research Ethics Committee of the Peking University Health Science Centre (IRB00001052-13082).

**Table 1:** The distribution and sample size of girls aged 9–18 among 24 ethnic minorities from 1985 to 2010

Ethnic minorities	Source province/region	Number (millions) (27)^a^	Minority population ranking^a^	1985	1991	1995	2000	2005	2010
Mongolian (urban)	Inner Mongolia	5.98	9	700	1,452	–	1,190	653	559
Mongolian (rural)	Autonomous Region			900	1,333	–	1,202	1,176	992
Mongolian (combined)				1,600	2,785	–	2,392	1,829	1,551
Hui (urban)	Ningxia Hui Autonomous	10.59	2	800	1,000	1,205	999	583	962
Hui (rural)	Region			694	1,000	1,193	1,000	1,462	1,185
Hui (combined)				1,494	2,000	2,398	1,999	2,045	2,147
Uighur (urban)	Xinjiang Uyghur	10.07	4	699	1,693	1,200	689	–	885
Uighur (rural)	Autonomous Region			700	1,700	1,200	312	–	1,008
Uighur (combined)				1,399	3,393	2,400	1,001	–	1,893
Kazakh (Hasake)		1.46	17	860	–	1,199	–	–	1,200
Kirghiz		0.19	31	449	–	1,200	500	–	1,225
Zhuang (urban)	Guangxi Zhuang	16.93	1	612	1,000	1,155	992	490	970
Zhuang (rural)	Autonomous Region			714	999	1,200	1,006	356	973
Zhuang (combined)				1,326	1,999	2,355	1,998	846	1,943
Yao		2.80	12	616	–	1,199	1,001	989	859
Korean (urban)	Jilin	1.83	14	800	998	1,199	981	395	1,034
Korean (rural)				1,100	984	1,198	989	474	1,117
Korean (combined)				1,900	1,982	2,397	1,970	869	2,151
Tibetan	Tibet Autonomous Region	6.28	8	769	994	1,197	1,144	999	1,047
Miao	Guizhou	9.43	5	696	982	1,101	1,720	999	992
Buyi	Guizhou	2.87	11	1,184	1,000	1,181	982	973	996
Dong	Guizhou	2.88	10	723	965	1,200	931	998	983
Tujia	Hunan	8.35	7	500	–	–	998	1,015	1,016
Bai	Yunnan	1.93	13	800	1,020	–	1,001	1,100	1,072
Hani	Yunnan	1.66	15	734	918	1,154	–	1,074	1,094
Dai	Yunnan	1.26	18	609	996	–	972	1,085	1,064
Lisu	Yunnan	0.70	20	728	–	1,106	–	1,087	1,027
Wa (Va)	Yunnan	0.43	24	613	–	1,090	–	1,098	1,076
Naxi (Nakhi)	Yunnan	0.33	26	700	1,018	1,200	–	1,096	1,094
Li	Hainan	1.46	16	700	999	1,200	909	–	865
Tu	Qinghai	0.29	28	1,103	583	600	1,001	1,059	1,085
Sala	Qinghai	0.13	34	224	574	600	999	1,051	1,072
Qiang	Sichuan	0.31	27	861	–	1,232	848	1,170	1,149
Yi	Sichuan	8.71	6	865	–	–	–	–	1,175
Total				21,739	23,186	27,145	24,319	21,382	31,711

a According to the Sixth National Census in 2010.

### Measures

The surveys used the *status quo* method to collect individual data on menarche. Girls aged ≥9 years in each CNSSCH were interviewed by a female physician or school nurse and asked whether or not they had begun to menstruate. Their responses were coded as *yes* (if they responded ‘yes, I have’) or *no* (if they responded ‘I haven't started yet’) (24). Because almost all schoolgirls of that age have some knowledge of menstrual periods from school health education, it was possible to obtain valid data on menarcheal status. The physicians or school nurses were well trained to explain menstruation to young girls, so that it could be distinguished from other phenomena, such as bleeding in the perineum due to injury. All the samples in the different cycles of the survey were independently collected and few participants were likely to have been selected repeatedly. Therefore, in testing the differences among different groups and between two consecutive years, a covariance of zero was assumed.

### Statistical analyses

The percentages of menstruating girls of each age by urban and rural subgroups were determined. We used probit analysis to determine the age at menarche for all girls. The probit analysis estimated the age and the 95% confidence interval (CI) at which 10, 25, 50, 75, and 90% of the girls reached menarche (25). The girls’ ages were recorded and calculated as decimal ages (e.g. 8.00–8.99 years, 9.00–9.99 years). In the probit analysis, the proportion of girls who had reached menarche was transformed into *y*, a normal equivalent deviate, i.e. P=*∫*^y^_–∞_f(θ)d(θ), where *y*=*a*+*bx*, *x* is the age in months and *a* and *b* were the parameters to be estimated from the data. Age at menarche was estimated when *p* was equal to 10, 25, 50, 75, and 90% (25, 26). The ages at these values of *p* are equivalent to the corresponding percentiles of ages at menarche, e.g. 50%=50th percentile age (the median age at menarche of the population). Differences in the percentages of menstruating girls among different ethnic minorities in 2010 were compared using the chi-square test, and differences in age at menarche between 1985 and 2010 survey points were tested by using z-test. A *p*-value less than 0.05 was considered as statistically significant. In order to facilitate the classification of ethnic minorities, age at menarche among the 24 ethnic minorities was examined by cluster analyses and cluster analyses adjusted for body mass index (BMI) were also conducted. All analyses were conducted with SPSS 20.0 (SPSS, Chicago, IL, USA).

## Results

### Age at menarche among 24 ethnic minorities in 2010

**Fig. 2:**
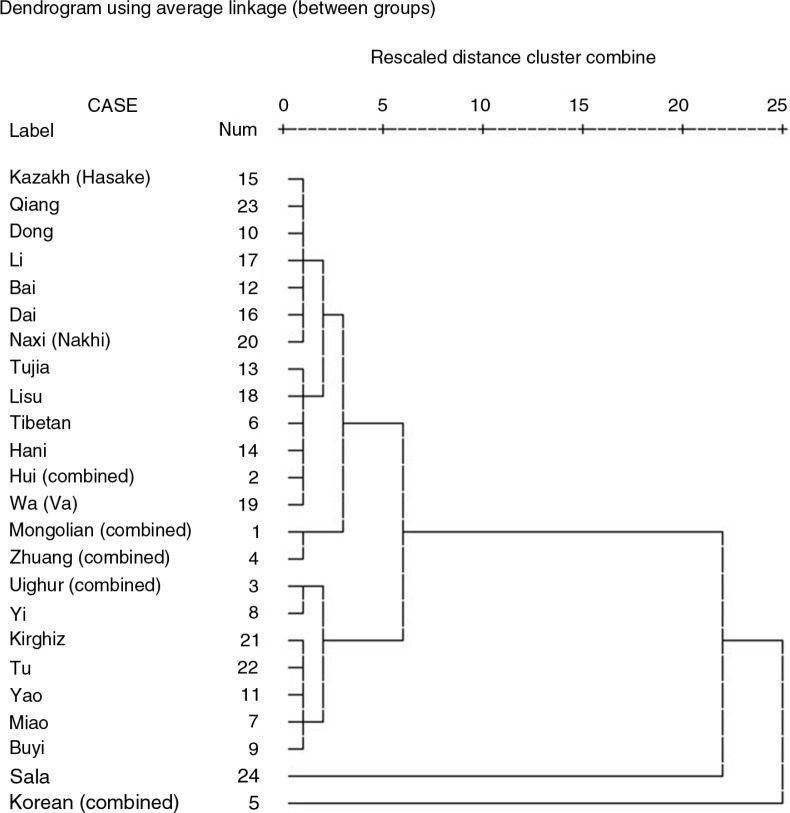
The clustering pattern of age at menarche among girls aged 9–18 from 24 ethnic minorities.

In 2010, only a small proportion of girls, including Tibetans (4.0%), Tu (1.0%), Mongolians (0.72%), Dai (0.91%), and Zhuang (0.52%), were menarcheal in the 9-year-old group. In the 12-year-old group, almost all ethnic minorities, except the Sala, were already menstruating. In the 13-year-old group, the percentage of menarche among the Sala was the lowest (32.17%), while that among the Korean girls was the highest (93.23%) (*χ* ^2^=810.11, *p<*0.01). By the age of 18, 99.67% of the ethnic minority girls were menstruating (Supplementary Table 1). The average ages at menarche among the three ethnic minorities with the earliest ages at menarche in 2010 were as follows: Koreans (11.79 years), Mongolians (12.44 years), and the Zhuang (12.52 years); those with the latest average ages at menarche were the Sala (14.32 years), Yi (13.74 years), and Uighurs (13.67 years). However, in 1985, the average ages at menarche of the three ethnic minorities with the earliest age at menarche were the Zhuang (13.21 years), Naxi (13.35 years), and Koreans (13.36 years) and the three ethnic minorities with the latest age at menarche were the Lisu (14.83 years), Kirghiz (14.72 years), and Kazakhs (14.54 years) (Table 2). The cluster analysis indicated that the 24 ethnic minorities in 2010 could be classified into three groups: Koreans were classified as the group having the youngest age at menarche; the Sala belonged to the group having the oldest age at menarche; and the other 22 ethnic minorities were classified in the middle group (Fig. 2). BMI showed an inverse correlation with age at menarche (*R*=−0.490) but, when age at menarche was adjusted for BMI in the cluster analysis, the classification of the various ethnic minority groups changed negligibly: Koreans and Mongolians were classified into the earlier age group, and the classifications of the other ethnic minorities were unchanged (Supplementary Fig. 1).

**Table 2:** Age at menarche (95% CI) of girls aged 9–18 among 24 ethnic minorities from 1985 to 2010

Ethnic minorities	1985	1991	1995	2000	2005	2010	Difference 1985 to 2010	Months of decline per decade
Mongolian (urban)	13.68 (13.50–13.87)	13.73 (13.32–14.15)	–	12.96 (12.76–13.44)	12.51 (12.02–12.94)	12.26 (12.06–12.46)	−1.42*	6.82
Mongolian (rural)	14.06 (13.90–14.25)	13.95 (13.56–14.35)	–	13.44 (12.00–14.66)	12.93 (12.76–13.09)	12.52 (12.36–12.68)	−1.54*	7.39
Mongolian (combined)	13.95 (13.82–14.07)	13.93 (13.53–14.35)	–	13.27 (12.47–14.01)	12.78 (12.51–13.04)	12.44 (12.03–12.84)	−1.51*	7.25
Hui (urban)	13.60 (13.42–13.79)	13.22 (12.98–13.46)	13.14 (12.92–13.35)	13.03 (12.88–13.25)	12.90 (12.70–13.10)	13.03 (12.46–13.56)	−0.57*	2.74
Hui (rural)	13.95 (13.83–14.07)	13.33 (13.02–13.65)	13.73 (13.53–13.94)	13.90 (13.47–14.30)	13.43 (13.12–13.73)	13.20 (11.88–14.68)	−0.75	3.60
Hui (combined)	13.73 (13.55–13.91)	13.38 (13.15–13.60)	13.46 (13.30–13.62)	13.44 (12.63–14.18)	13.28 (13.00–13.54)	13.09 (12.42–13.76)	−0.64*	3.07
Uighur (urban)	14.21 (14.04–14.38)	14.03 (13.84–14.22)	13.57 (13.36–13.78)	13.47 (13.26–13.72)	–	13.56 (12.92–14.12)	−0.65*	3.12
Uighur (rural)	14.44 (14.25–14.63)	14.29 (14.03–14.54)	14.49 (14.28–14.70)	13.48 (13.29–13.77)	–	13.78 (13.40–14.14)	−0.66*	3.17
Uighur (combined)	14.42 (14.29–14.54)	14.17 (14.00–14.35)	13.92 (13.77–14.08)	13.48 (13.30–13.80)	–	13.67 (13.34–13.99)	−0.75*	3.60
Kazakh (Hasake)	14.54 (14.39–14.69)	–	13.82 (13.64–14.02)	–	–	12.85 (12.70–13.00)	−1.69*	8.11
Kirghiz	14.72 (14.49–14.95)	–	14.65 (14.44–14.86)	13.86 (13.67–14.03)	–	13.40 (12.81–13.96)	−1.32*	6.34
Zhuang (urban)	13.35 (13.18–13.53)	12.97 (12.64–13.30)	13.11 (12.92–13.30)	12.58 (11.90–13.22)	13.02 (12.69–13.33)	12.26 (12.11–12.41)	−1.09*	5.23
Zhuang (rural)	13.20 (12.97–14.43)	13.12 (12.58–13.39)	14.68 (14.41–14.96)	13.12 (12.20–13.89)	12.42 (11.84–12.99)	12.79 (12.30–13.28)	−0.41*	1.97
Zhuang (combined)	13.21 (13.05–13.36)	13.02 (12.83–13.21)	13.83 (13.66–14.00)	12.86 (12.52–13.18)	12.71 (12.38–13.04)	12.52 (12.33–12.70)	−0.69*	3.31
Yao	14.43 (14.22–14.64)	–	13.59 (13.38–13.79)	13.54 (13.24–13.95)	12.67 (11.75–13.52)	13.41 (12.81–13.99)	−1.02*	4.90
Korean (urban)	13.22 (13.07–13.38)	12.92 (12.69–13.15)	12.92 (12.67–13.16)	12.30 (12.07–12.61)	12.25 (11.93–12.56)	11.94 (11.71–12.16)	−1.28*	6.14
Korean (rural)	13.56 (13.39–13.73)	13.34 (12.99–13.69)	12.50 (12.28–12.72)	12.87 (12.54–13.19)	12.43 (13.32–12.54)	11.65 (11.32–11.97)	−1.91*	9.17
Korean (combined)	13.36 (13.25–13.47)	13.14 (12.93–13.35)	12.75 (12.58–12.92)	12.59 (12.21–12.96)	12.35 (12.21–12.49)	11.79 (11.68–11.89)	−1.57*	7.54
Tibetan	13.63 (13.42–13.84)	13.73 (13.35–14.13)	13.72 (13.52–13.92)	13.04 (12.72–13.39)	13.42 (12.84–14.00)	13.12 (11.41–14.81)	−0.51	2.45
Miao	13.71 (13.53–13.89)	13.15 (12.78–13.54)	13.54 (13.33–13.75)	12.95 (12.43–13.39)	13.00 (12.83–13.18)	13.36 (12.99–13.72)	−0.35	1.68
Buyi	13.91 (13.68–14.13)	13.57 (13.15–14.01)	13.37 (13.08–13.66)	13.25 (12.93–13.69)	13.12 (10.70–15.60)	13.26 (13.01–13.51)	−0.65*	3.12
Dong	14.26 (14.07–14.45)	13.21 (12.95–13.47)	13.50 (13.31–13.68)	13.41 (13.08–13.80)	13.32 (13.16–13.48)	12.91 (12.48–13.34)	−1.35*	6.48
Tujia	14.05 (13.90–14.20)	–	–	13.63 (13.47–13.78)	11.90 (11.36–12.40)	13.02 (12.27–13.72)	−1.03*	4.94
Bai	13.91 (13.73–14.09)	13.15 (12.89–13.24)	–	13.44 (13.07–13.84)	12.89 (11.85–13.89)	12.79 (11.77–13.74)	−1.12*	5.38
Hani	14.42 (14.25–14.58)	17.17 (15.73–18.84)	13.40 (13.24–13.57)	–	13.37 (12.88–13.84)	13.12 (12.46–13.78)	−1.30*	6.24
Dai	14.27 (14.12–14.43)	13.43 (13.12–13.74)	–	13.39 (13.09–13.72)	13.24 (13.08–13.39)	12.73 (12.47–12.98)	−1.54*	7.39
Lisu	14.83 (14.66–14.99)	–	13.10 (12.88–13.34)	–	13.60 (13.43–13.76)	13.04 (12.39–13.68)	−1.79*	8.59
Wa (Va)	13.85 (13.63–14.08)	–	12.56 (12.34–12.78)	–	13.17 (11.77–14.58)	13.06 (12.91–13.20)	−0.79*	3.79
Naxi (Nakhi)	13.35 (13.20–13.51)	13.09 (12.88–13.31)	13.04 (12.86–13.22)	–	11.97 (11.83–12.11)	12.64 (12.48–12.79)	−0.71*	3.41
Li	13.10 (12.94–13.26)	13.02 (12.79–13.24)	13.04 (12.84–13.24)	13.23 (12.87–13.56)	–	12.95 (12.80–13.09)	−0.15	0.72
Tu	14.22 (14.02–14.43)	14.34 (13.83–14.86)	14.03 (13.80–14.26)	13.63 (13.46–13.81)	13.61 (12.89–14.33)	13.40 (12.76–13.99)	−0.82*	3.94
Sala	14.47 (14.18–14.76)	14.45 (14.09–14.81)	14.26 (13.87–14.67)	14.07 (13.82–14.31)	14.30 (14.15–14.44)	14.32 (14.02–14.61)	−0.15	0.72
Qiang	14.07 (13.91–14.23)	–	13.81 (13.63–13.99)	12.49 (11.67–13.11)	13.42 (13.28–13.56)	12.85 (12.71–13.00)	−1.22*	5.86
Yi	13.80 (13.60–14.01)	–	–	–	–	13.74 (13.47–14.00)	−0.06	0.29

* *p<*0.05 CI, confidence interval.

### Secular trends in age at menarche among 24 ethnic minorities from 1985 to 2010

**Fig. 3:**
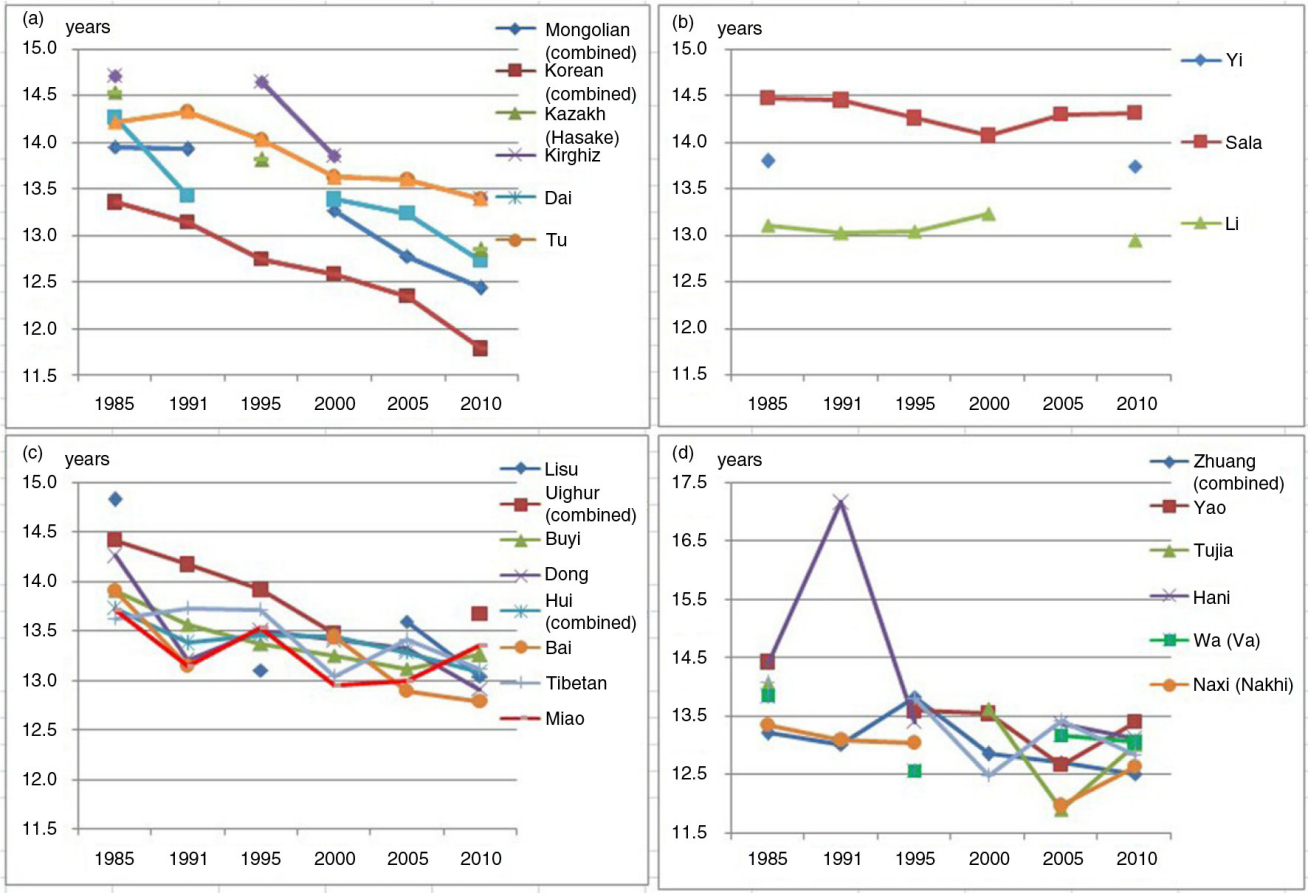
The secular trends regarding age at menarche of girls aged 9–18 among 24 ethnic minorities from 1985 to 2010. (a) The clearly declining trend over time of the Mongolians, Koreans, Kazakhs (Hasake), Kirghiz, Dai, and Tu; (b) the relatively flat trend of the Yi, Sala, and Li; (c) the decreasing trend but with small upward fluctuations of less than 0.5 years displayed by the Lisu, Uighurs, Buyi, Dong, Hui, Bai, Tibetans, and Miao; and (d) the decreasing trend but with large upward fluctuations of more than 0.5 years exhibited by the Zhuang, Yao, Tujia, Hani, Wa (Va), Naxi (Nakhi), and Qiang.

From 1985 to 2010, the age at menarche showed a downward shift in each of the 24 ethnic minorities, but with different patterns of decline. Some minority groups, such as the Mongolians, Koreans, Kazakhs (Hasake), Kirghiz, Dai, and Tu, showed a clearly declining trend over time.; some minority groups, such as the Yi, Sala, and Li, showed a relatively flat trend with no statistically significant difference over time, whereas the remainder showed a decreasing trend with upward fluctuations over time (Fig. 3). As a result, the Lisu, Kazakhs, and Koreans showed the largest reductions, with age at menarche found to be 1.79 (*p<*0.05), 1.69 (*p<*0.05), and 1.57 (*p<*0.05) years earlier, respectively, in 2010 than in 1985. In contrast, the Yi, Sala, and Li showed the smallest reductions, with age at menarche only 0.06 (*p>*0.05), 0.15 (*p>*0.05), and 0.15 (*p>*0.05) years earlier, respectively, in 2010 than in 1985. Among Mongolian, Hui, Uighur, Zhuang, and Korean girls, the age at menarche was earlier in urban girls than in rural girls at each survey point except for Korean girls in 2010, where both urban and rural groups showed a similar decreasing trend over time (Table 2).

## Discussion

Age at menarche reflects numerous health aspects of a population (11, 12), and may also represent a potential health risk if it occurs earlier than the norm. The results presented in this study demonstrated large variations in age at menarche among different ethnic minorities. Compared to the other ethnic minorities in our study, Korean girls had the earliest age at menarche (11.79 years in 2010). About 76.39% of the girls of Korean ethnicity in primary schools (up to 12 years of age) were menstruating in 2010, whereas no girls of Sala ethnicity in this range were menstruating. There are many factors influencing age at menarche, such as genetics, geography, nutritional habits, exercise (11), and even climate. Dossus et al. (28) found that women born at lower latitudes or in regions with a higher annual or spring/summer ultraviolet radiation (UVR) dose had their menarche 3 to 4 months earlier than women born at higher latitudes or in regions with lower UVR doses. The household's economic conditions also play an important role (12). Gustafsson and Sai (29) found that ethnic minority villages in northeast China had a somewhat better economic situation than the average majority village, in contrast to minority villages in the southwest where considerably poorer economic circumstances were found (29, 30). Thus, better economic conditions could partly explain the earlier age at menarche of Korean girls (who lived in northeast China) compared to other ethnic minority girls. Nevertheless, the influence of ethnicity is likely due to a complex interplay of factors that need to be studied further.

The present study is the first to demonstrate the trend of declining age at menarche in Chinese ethnic minority girls. Globally, the trend of age at menarche varies greatly between countries and regions and has been characterized by about a 3- to 4-month decrease per decade from 1830 to 1980 in Western Europe and approximately 2.5 months decrease per decade from 1963–1970 to 1988–1994 in the United States (1). The rate of decline in age at menarche has recently slowed in Japan, the Netherlands, Germany, and Bulgaria (3, 7, 31) and has remained stable in Belgium and Norway (7). In China, from 1985 to 2010, the average age at menarche showed a downward shift, and this decrease has been approximately 4.5 months per decade among Han girls (10).

In the present study, we found that the occurrence of a downward secular trend in age at menarche was evident in each of the 24 ethnic minorities, but to different degrees. When compared with Han girls, the 24 ethnic minority groups could be subdivided into three categories: 1) ethnic minority girls whose differences in age at menarche from 1985 to 2010 were larger than Han girls, i.e. Mongolians, Lisu, Kazakhs (Hasake), Koreans, Dai, Dong, Kirghiz, Hani, Qiang, Bai, Tujia, and Yao; 2) ethnic minority girls whose differences in age at menarche from 1985 to 2010 were smaller than Han girls, i.e. Tu, Wa (Va), Uighur, Naxi (Nakhi), Zhuang, Buyi, and Hui; and 3) ethnic minority girls whose differences in age at menarche from 1985 to 2010 were non-significant, such as the Yi, Sala, Li, Tibetans, and Miao.

The pattern of results thus indicated that not all ethnic minorities showed a significant decline in age at menarche during the years in question, suggesting that differences in secular trends among ethnic minorities may well be related to specific features of certain ethnic minority groups. Thus, even among ethnic minorities from the same provinces or regions, large variations in age at menarche were observed. For example, the Dong, Buyi, and Miao are all from Guizhou Province; however, they showed different patterns concerning age at menarche trends: Dong girls (6.48 months per decade) had larger differences regarding average age at menarche than Han girls, Buyi girls (3.12 months per decade) had smaller differences than Han girls, whereas the Miao (1.68 month per decade) had no significant differences over the past 25 years. Similarly, in Yunnan Province, the Bai, Hani, Dai, Lisu, Wa (Va), and Naxi (Nakhi) also showed different patterns of declining menarcheal age. However, the Kazakhs (Hasake) and Kirghiz are both from Xinjiang Uyghur Autonomous Region, and both showed clearly declining trends of age at menarche. The results indicated that cultural lifestyles, socio-economic status, and diet (fat intake) might be key factors that vary among different minorities even when they are residing in the same regions.

With regard to the same ethnic minority residing in different regions, we found that both urban and rural girls from the same ethnic minority showed similarly declining trends. However, the age at menarche was earlier in urban girls than their rural counterparts for most ethnic minorities, possibly due to environmental factors. One potential environmental influence is the rapid economic development in China from a poor developing country with a GDP of only US$60 per capita in 1978 to a middle income country with a GDP of US$4,700 per capita in 2010 (exchange rate: US$1= RMB¥6.35) (32). Furthermore, since 1978, socio-economic conditions have always been better in urban than rural areas. For example, the per capita annual disposable income has been higher in urban than rural households and the Engel coefficient has been lower in urban than rural households (33).

Consistent with the results of South Korean research, Korean girls in China had a declining trend in age at menarche with 7.54 months decrease per decade. A study by Hwang found that the mean menarcheal age decreased from 16.8 to 12.7 years, corresponding to –0.64 years (approximately –7.68 months) per decade among a total of 1,061 South Korean women born between 1920 and 1986 from Ansan Cohort Study samples and separate schoolgirl samples (34). Furthermore, the 2005 Korean National Health and Nutrition Survey also found that the mean age at menarche decreased from 16.90 years for women born between 1920 and 1925 to 13.79 years for those born between 1980 and 1985, indicating a downward trend of 0.68 years (approximately 8.16 months) per decade in age at menarche (35).

Several studies found that the downward shifts of the age at menarche have been accompanied by a simultaneous increase in BMI (10, 36, 37). Increased adiposity may trigger estrogen production, leading to the early onset of puberty in girls (36). A cohort study by Frisch also found that weight and body fat might be causal determinants of age at menarche (38). However, other studies found that age at menarche may be an indicator of susceptibility to overweight and obesity in adulthood (37). The studies in South Korea also found that early menarche is a risk factor for obesity in adults or inversely associated with various forms of dysglycemia; thus, early menarche represents a potential health risk (39, 40). Epidemiologic evidence supports the conclusion that earlier menarche is associated with detrimental health effects: women who experience earlier menarche are more likely to have estrogen-dependent diseases such as breast cancer (15) and also experience earlier mortality than their peers (41). Since earlier menarche and higher BMI acting together may pose a risk for women's health in later years, changes are required in sexual education in primary schools in order to reach the younger age groups. Health education and interventions such as obesity prevention should be designed and facilitated for girls with early menarche in order to decrease their likelihood of future health risks.

In contrast to the ethnic minorities that had shown considerable declining trends of age at menarche, the differences in age at menarche over the past 25 years among the Yi, Sala, Li, Tibetan, and Miao minorities were too small to be statistically significant. Tibetan minorities live at high altitudes, where the age at menarche is delayed. A study conducted in a Tibetan-speaking population living at 3,250–3,560 meters in Nepal found that the median age at menarche was 16.2 years in the 1980s (42). However, the age at menarche of Tibetan girls in China was significantly younger than that population and was closer to that of the Miao, although they belong to different language groups (Tibeto-Burman and Miao-Yao, respectively), according to linguists (43). Nevertheless, other ethnic minorities living in the same regions or areas showed different patterns than the Yi, Sala, Li, and Miao, and the underlying reasons for these ethnic differences need to be investigated further.

Other ethnic minorities such as the Mongolians, Lisu, Kazakhs (Hasake), Dai, Dong, Kirghiz, Hani, Qiang, Bai, Tujia, Yao, Tu, Wa (Va), Uighurs, Naxi (Nakhi), Zhuang, Buyi, and Hui all showed a declining trend in age at menarche. Of these, Mongolian girls not only showed a clearly declining trend of 7.25 months decrease per decade, but the menarcheal age was also 0.14 years earlier than that of the Han girls from the Inner Mongolia Autonomous Region in 2010, possibly attributable to differing diet and nutritional habits (44). These results indicate that Mongolian girls, similar to Korean girls, also need health education about reproductive health. Although the ages at menarche among most ethnic minorities are in the middle range regarding age group and are not earlier than that of Han girls from the same province or region (44), ethnic minority girls would in general benefit from health education in areas such as physiology, nutrition, and the health benefits of physical activity, although the emphasis on particular topics may vary according to the specific minority.

Our study has several limitations. Firstly, it was not a prospective cohort study, because each CNSSCH was a cross-sectional survey conducted with different participants. The average age at menarche might not reflect the exact situation in the population; this can be clarified only by a longitudinal cohort study. Secondly, information was not obtained about the actual menarche dates of the girls. However, the *status quo* method – when used in a large study population such as the current sample – is considered to be even more reliable than the recall method for obtaining menarche dates. Thirdly, the sample size of some ethnic minorities, for example, the Sala, may have been inadequate and larger surveys are needed in the future. In addition, previous findings concerning the age at menarche in ethnic minorities such as the Mongolians, Uighurs, Kazakhs, Miao, and Yao are limited, and thus, comparative studies across different countries or regions are not available.

## Conclusion

Age at menarche varied widely among different ethnic minorities. From 1985 to 2010, the age at menarche among Chinese ethnic minority girls declined significantly among 19 of 24 ethnic minorities included in the current study. During that period, 12 ethnic minorities showed clearly declining rates, with a decrease of over 4.5 months per decade, which is a larger decrease than that shown among Han girls. Seven ethnic minorities showed decreasing trends and five ethnic minorities showed no significant differences. The age at menarche of Korean girls was significantly earlier than other ethnic minorities, and their secular trend was in accordance with results from women living in South Korea. The overall pattern of results suggests that interventions and health education should be designed to meet the specific needs of different ethnic minorities. For example, education concerning early menarche should be an important part of health education for Korean girls but is not necessarily as urgently indicated among Sala girls.

## Supplementary Material

Secular trends in age at menarche among Chinese girls from 24 ethnic minorities, 1985 to 2010
