# Supplementary material for: Secular trends in age at menarche among Chinese girls from 24 ethnic minorities, 1985 to 2010
Source: Glob Health Action. 2015 Jul 27;8:10.3402/gha.v8.26929. doi: 10.3402/gha.v8.26929 (PMC4518164; doi:10.3402/gha.v8.26929)
Supplement: Secular trends in age at menarche among Chinese girls from 24 ethnic minorities, 1985 to 2010 [file GHA-8-26929-s001.doc]

Table S1 Proportion of girls with menarche by age and ethnic minority in 2010

| Ethnic minorities | 9- | 10- | 11- | 12- | 13- | 14- | 15- | 16- | 17- | 18- | Total |
| --- | --- | --- | --- | --- | --- | --- | --- | --- | --- | --- | --- |
| Mongolian | 0.72 | 0.70 | 22.64 | 51.97 | 79.49 | 96.20 | 99.43 | 100.00 | 99.39 | 100.00 | 67.63 |
| Hui | 0.00 | 15.83 | 2.26 | 25.63 | 62.20 | 72.05 | 90.65 | 100.00 | 100.00 | 99.53 | 55.89 |
| Uighur | - | 0.00 | 1.20 | 2.09 | 51.88 | 86.50 | 91.25 | 100.00 | 100.00 | 100.00 | 67.35 |
| Kazak | 0.00 | 1.72 | 7.26 | 43.90 | 74.59 | 88.89 | 95.93 | 99.16 | 100.00 | 100.00 | 61.08 |
| Kirgiz | 0.00 | 0.74 | 0.00 | 5.44 | 79.25 | 90.48 | 91.07 | 99.17 | 100.00 | 100.00 | 54.37 |
| Zhuang | 0.52 | 1.49 | 21.61 | 44.10 | 75.90 | 97.41 | 100.00 | 100.00 | 100.00 | 100.00 | 63.92 |
| Yao | 0.00 | 3.33 | 4.71 | 10.00 | 48.91 | 86.96 | 97.98 | 100.00 | 100.00 | 100.00 | 61.23 |
| Korean | 0.00 | 8.06 | 40.93 | 76.39 | 93.23 | 100.00 | 99.49 | 99.54 | 99.17 | 100.00 | 72.99 |
| Tibetan | 4.00 | 4.50 | 5.71 | 13.21 | 52.73 | 96.80 | 98.15 | 100.00 | 99.05 | 100.00 | 59.12 |
| Miao | 0.00 | 3.09 | 5.00 | 24.00 | 57.00 | 67.00 | 98.99 | 100.00 | 100.00 | 100.00 | 55.65 |
| Buyi | 0.00 | 3.00 | 8.00 | 19.19 | 62.63 | 78.00 | 95.00 | 100.00 | 100.00 | 100.00 | 56.53 |
| Dong | 0.00 | 5.00 | 12.36 | 16.33 | 77.78 | 87.88 | 100.00 | 100.00 | 100.00 | 100.00 | 60.43 |
| Tujia | 0.00 | 3.80 | 19.80 | 20.19 | 65.05 | 91.51 | 92.66 | 99.06 | 100.00 | 97.12 | 61.02 |
| Bai | 0.00 | 0.00 | 22.94 | 26.61 | 77.27 | 88.18 | 100.00 | 100.00 | 100.00 | 99.05 | 62.41 |
| Hani | 0.00 | 2.73 | 3.64 | 6.36 | 80.91 | 90.91 | 98.15 | 100.00 | 100.00 | 100.00 | 58.04 |
| Dai | 0.91 | 0.91 | 16.51 | 45.87 | 72.73 | 90.91 | 97.27 | 97.27 | 99.09 | 100.00 | 61.00 |
| Lisu | 0.00 | 13.73 | 7.55 | 22.43 | 53.27 | 89.81 | 97.20 | 99.08 | 100.00 | 99.09 | 61.34 |
| Wa | 0.00 | 0.00 | 1.82 | 34.86 | 68.54 | 87.27 | 100.00 | 100.00 | 100.00 | 100.00 | 59.01 |
| Naxi | 0.00 | 0.00 | 19.27 | 54.55 | 67.89 | 90.91 | 99.09 | 100.00 | 100.00 | 100.00 | 63.16 |
| Li | 0.00 | 0.00 | 8.33 | 22.73 | 77.45 | 94.03 | 97.70 | 100.00 | 97.85 | 96.67 | 68.67 |
| Tu | 1.00 | 0.00 | 0.00 | 25.23 | 60.82 | 81.36 | 90.27 | 98.11 | 100.00 | 100.00 | 56.31 |
| Sala | 0.00 | 0.00 | 0.00 | 0.00 | 32.71 | 57.94 | 75.00 | 99.11 | 100.00 | 100.00 | 44.87 |
| Qiang | 0.00 | 1.83 | 15.00 | 33.63 | 67.26 | 88.98 | 100.00 | 100.00 | 100.00 | 100.00 | 60.66 |
| Yi | 0.00 | 0.00 | 3.39 | 10.74 | 50.00 | 76.52 | 85.59 | 96.52 | 100.00 | 99.15 | 51.74 |


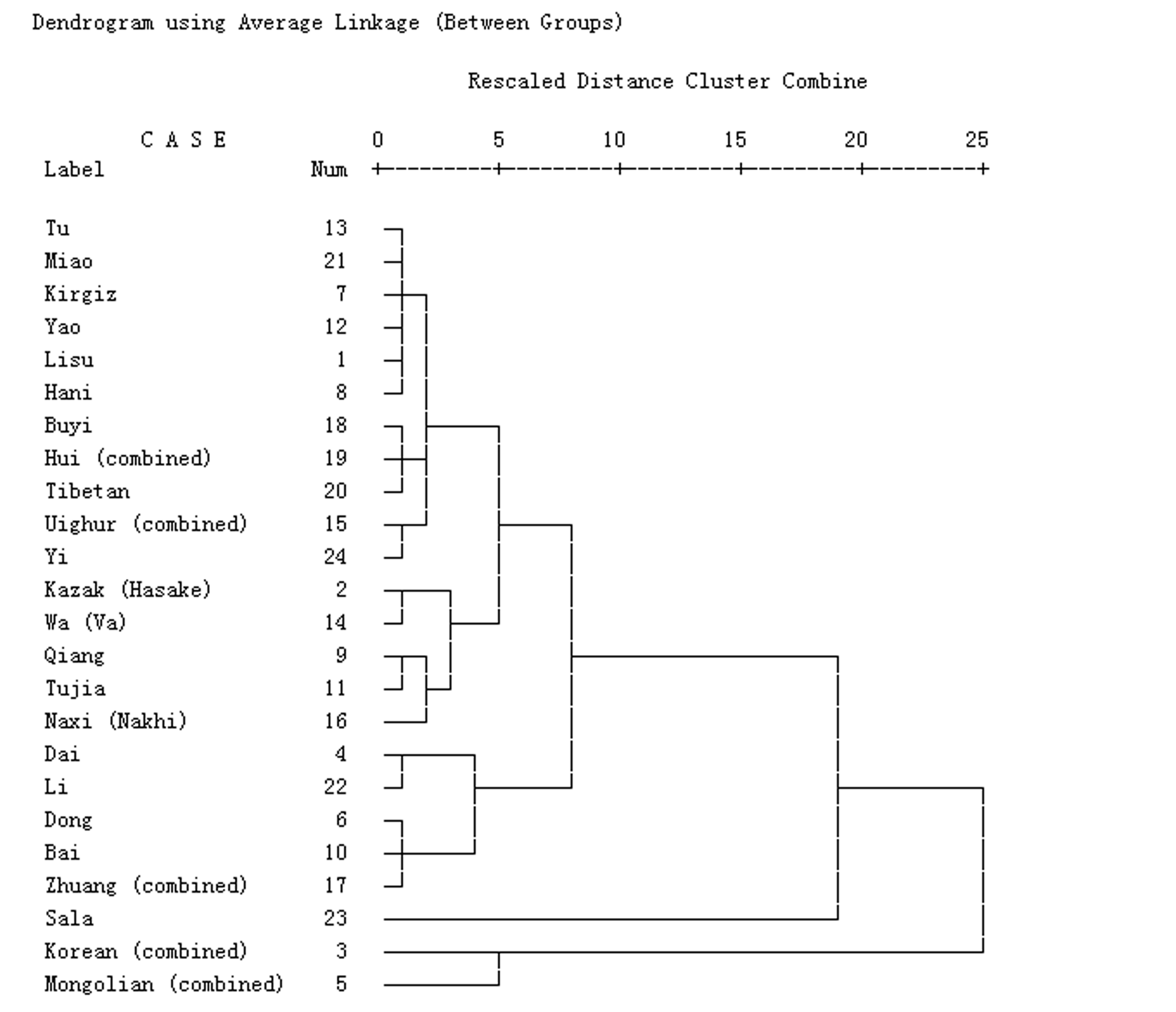


Figure S1 The clustering pattern of age at menarche adjusted by BMI among girls aged 9 to 18 from 24 ethnic minorities.
